# Generation of new cisgenic apple lines resistant to either apple scab or fire blight

**DOI:** 10.1007/s00425-026-05051-6

**Published:** 2026-06-24

**Authors:** Ina Schlathölter, Henryk Flachowsky, Bruno Studer, Henk J. Schouten, Andrea Patocchi, Giovanni A. L. Broggini

**Affiliations:** 1 Department of Plant Breeding, Breeding Research, Agroscope, Mueller-Thurgau-Strasse 29, 8820 Waedenswil, Switzerland; 2 Molecular Plant Breeding, Institute of Agricultural Sciences, ETH Zurich, Universitaetstrasse 2, 8092 Zurich, Switzerland; 3 Julius Kühn-Institut (JKI)-Federal Research Centre for Cultivated Plants, Institute for Breeding Research On Fruit Crops, Pillnitzer Platz 3a, 01326 Dresden-Pillnitz, Germany; 4 Department of Plant Breeding, Wageningen University & Research, Droevendaalsesteeg 1, 6708PB Wageningen, The Netherlands; 5 Department of Plant Breeding, Fruit Breeding Group, Agroscope, Mueller-Thurgau-Strasse 29, 8820 Waedenswil, Switzerland; 6 Present Address: Department of Plant Pathology, University of Florida, Gainesville, FL 32611 USA

**Keywords:** *Malus domestica*, Cisgenesis, Breeding, *Erwinia amylovora*, *Venturia inaequalis*

## Abstract

**Main conclusion:**

Cisgenic introduction of *Rvi15* or *FB_MR5* into ‘Gala’ generated single-copy lines with ≤ 201 bp vector remnants and high disease resistance, supporting cisgenesis as a powerful tool for commercial apple improvement.

**Abstract:**

Cisgenesis can be used to transfer disease resistance genes from wild apple germplasm into established apple cultivars, reducing their susceptibility to diseases. This approach allows combining disease resistance and fruit quality traits without compromising the desirable characteristics of the original cultivar. In our study, we used the binary plasmid vector pMF1 to create three apple scab-resistant cisgenic ‘Gala’ lines with the apple scab resistance gene *Rvi15*, and three fire blight-resistant cisgenic 'Gala' lines with the fire blight resistance gene *FB_MR5*. Quantitative PCR revealed that five of six cisgenic lines had a single integrated T-DNA copy, and targeted locus amplification enabled the identification and characterization of insertion sites in five lines. All characterized lines showed T-DNA trimming at the borders, with a total length of non-endogenous sequences at their respective insertion sites ranging from 135 to 201 base pairs. The presence of such residual vector sequences can influence the regulatory status of cisgenic plants, as some countries may classify these lines as genetically modified plants. All cisgenic lines exhibited increased disease resistance compared to the untransformed control genotype ‘Gala’. However, the achieved resistances are based on single genes, and it will be necessary to develop combinations of different resistance genes for more durable resistance. Resistance, durability, and evolving regulatory frameworks are crucial factors that together will determine the success of the cisgenic approach in achieving both high fruit quality and resistance for sustainable apple production.

**Supplementary Information:**

The online version contains supplementary material available at 10.1007/s00425-026-05051-6.

## Introduction

Apple (*Malus domestica* Borkh.) production is threatened by several diseases. While chemical control is effective against fungal pathogens, e.g., *Venturia inaequalis* it can be ineffective against *Erwinia amylovora*, the bacterial pathogen causing fire blight. The most sustainable approach to reducing crop losses and chemical inputs is the use of disease-resistant cultivars. However, most commercially successful apple cultivars are susceptible to diseases, and introgressing major disease resistance genes from wild apple genotypes requires time and extensive breeding efforts (Schlathölter et al. 2018) and will always lead to the development of novel cultivars with new characteristics.

Cisgenesis enables increasing disease resistance in an established cultivar without compromising its desirable characteristics. This is achieved using genetic modification techniques to amend established cultivars with resistance genes (including their native regulatory sequences) from sexually compatible donors (Schouten et al. 2006). Cisgenesis in apple was achieved by *Agrobacterium*-mediated plant transformation using either binary vectors pMF1 (Schaart et al. 2004) or p9-Dao-FLPi (Würdig et al. 2015). These vectors carry different inducible recombinase systems, allowing the excision of non-endogenous sequences required for the generation process (e.g., antibiotic resistance for selection) but unwanted in the final product. Most transgenic or vector-derived sequences are removed when the recombination system is activated in a transgenic intermediate line. However, recombination often leaves some non-endogenous sequences in the final product, including the left and right T-DNA borders, a recombinase recognition site, and spacer sequences used for cloning. The length of integrated non-endogenous sequences after recombination is expected to be greater for vector p9-Dao-FLPi than for pMF1. When cloning the fire blight resistance gene *FB_MR5* from the wild apple *Malus* x *robusta* 5 into both vectors, the non-endogenous sequence between the left border (LB) and *FB_MR5* is approximately 420 bp in p9-Dao-FLPi and 169 bp in pMF1. At the right border (RB), these lengths are 116 bp and 60 bp, respectively (Kost et al. 2015; Schaart et al. 2004; Würdig et al. 2015). Despite this, the extent of non-endogenous sequences in the final cisgenic line can vary; it can be larger or smaller than expected. It may be larger, because vector-derived sequences flanking the T-DNA could also be unexpectedly integrated due to a leaky T-DNA LB, leading in some cases to the incorrect excision of the T-DNA and resulting in the integration of part of the transformation plasmid backbone, as observed using both vectors (Kost et al. 2015; Vanblaere et al. 2014). It may be smaller because T-DNA extremities can be trimmed during integration, resulting in T-DNA RB and/or LB truncation or absence at insertion sites (Dalla Costa et al. 2020). The extent of non-endogenous sequences detectable in the final product could become a decisive criterion if future regulatory frameworks differentiate categories of genetically modified (GM) plants, regardless of the biosafety risks associated with such sequences.

The cisgenic approach improved the resistance of the cultivar ‘Gala’ against the most problematic diseases in apple orchards. Resistance to apple scab was conferred by *Rvi6* (Joshi et al. 2011; Krens et al. 2015; Vanblaere et al. 2011, 2014; Würdig et al. 2015), while fire blight resistance was conferred by *FB_MR5* (Kost et al. 2015). This latter cisgenic line C44.4.146 was investigated in a field trial over 5 years (Schlathölter et al. 2021, 2022, 2023). The described cisgenic lines were generated for research, not commercial release or apple production. Neither resistance gene is durable in the field, as shown by the reported emergence of pathogen strains able to overcome these major resistance genes (Parisi et al. 1993; Vogt et al. 2013). While no other fire blight resistance genes have been cloned and validated, the apple scab resistance genes *Rvi15* from GMAL2473 (Schouten et al. 2014) and *Rvi12* Hansen’s baccata #2 (Yousaf et al. 2025) were cloned. No virulent strains were able to grow on GMAL2473, the differential host used to monitor *Rvi15*, while rare events of resistance breakdown to *Rvi12* were observed within the international monitoring project on apple scab virulence VINQUEST (Patocchi et al. 2020). However, Peil et al. (2023) showed that *Rvi4* and *Rvi15* are the same gene, and virulent strains have already been reported for *Rvi4* (category “sometimes overcome”, Patocchi et al. 2020).

This work aims to generate *FB_MR5* fire blight or *Rvi15* apple scab-resistant cisgenic apple lines using the pMF1 vector and their functional and molecular characterization. The molecular analysis focuses on the characterization of the insertion sites, the extent of non-endogenous sequences at T-DNA-genome junctions, and the consistency of the recombinase-based excision system of the pMF1 vector.

## Materials and methods

### Vectors, plant material, and cisgenic process

The binary vector pMF1::*FB_MR5* was purchased from DNA Cloning Service e.K. (Hamburg, DE). It was obtained by cloning the *FB_MR5* resistance gene with 1995 bp of 5’-UTR and 1,547 bp of 3’-UTR into the vector pMF1 (Suppl. Fig. S1).

The in vitro ‘Gala Galaxy’ apple line, kindly provided by Dr. Elisabeth Chevreau (INRAe, Angers, FR), and previously used for generating the cisgenic line C44.4.146 (Kost et al. 2015; Schlathölter et al. 2023), was used for transformation. In vitro cultures of the transgenic apple lines 603 and 604, generated using the pMF1::*Rvi15* (*Vr2-C*) vector for transformation of a different in vitro ‘Gala’ line (Schouten et al. 2014), were also used. Additionally, transgenic lines 605 and 606, generated but not reported in the same study, were provided and included in this work. For clarity, a "t” or a “c” was added in front of the transgenic event number (e.g., t603) to distinguish them from the derived cisgenic lines, respectively.

Plant transformation using the pMF1::*FB_MR5* binary vector was performed as described by Vanblaere et al. (2011). DNA was extracted from leaves of regenerated transgenic plantlets and tested by PCR to confirm the presence of the T-DNA and absence of vector backbone sequences using primers IPCR_codA_1 and pmf_bb2 (Vanblaere et al. 2014). The cisgenic lines were regenerated from intermediate transgenic lines (carrying *FB_MR5* or *Rvi15*), following induction of the recombinase-R by dexamethasone treatment as described by Vanblaere et al. (2011), causing an excision of the unwanted transgenic sequences. Regenerants were maintained on elongation medium and tested for the absence of the gene *NptII*, present on the excisable transgenic cassette, as described by Vanblaere et al. (2011). From each transgenic line, a single cisgenic plant was regenerated and investigated, except for t603, where five independent cisgenic regenerants (cisgenic sublines c603.1-c603.5) were used to investigate the consistency of the recombination process. For all other analyses, only the first regenerant (c603.1, referred to as c603) was considered. Cisgenic lines were acclimatized to greenhouse conditions by micrografting onto apple seedlings (Joshi et al. 2011), grown to a shoot length of approximately 1 m, and multiplied by grafting on M9 T337 rootstocks to produce the replicates necessary for the disease resistance tests and further molecular characterization.

### Copy-number quantification by qPCR

Two-to-three single leaves were collected from plants grown in the greenhouse for each investigated genotype and freeze-dried. The plant tissue was mechanically homogenized with glass beads using a Savant Bio 101 FastPrep cell homogenizer (Savant, Holbrook, NY, USA). DNA was extracted using the DNeasy Plant Mini Kit (Qiagen, Düsseldorf, Germany) and quantified on a Nanodrop spectrophotometer (Thermo Fisher Scientific Inc., Waltham, USA).

The copy number of the resistance gene integration events in the cisgenic lines was determined by qPCR using specific TaqMan assays for each resistance gene. The elongation factor gene *EF1α* served as a reference gene (Gusberti et al. 2012). The assay developed by Kost et al. (2015) was used for *FB_MR5*. A new TaqMan assay was developed for *Rvi15*, using the primers Rvi15q1 forward and reverse (each at a final concentration of 900 nM), and the *Rvi15* TaqMan probe at a final concentration of 250 nM. Primer and probe sequences are provided in Suppl. Table S1. The qPCR mix contained 3 µl DNA in a 10 µl reaction with TaqMan Fast Universal Master Mix (Thermo Fisher Scientific Inc., Waltham, USA). The reaction was performed on a 7500 Fast Real-Time PCR system (Thermo Fisher Scientific Inc., Waltham, USA) using the following conditions: 20 s at 95 °C, followed by 40 cycles of 3 s at 95 °C and 60 s at 60 °C. DNA of the cisgenic line C44.4.146, known to carry a single copy of *FB_MR5* (Kost et al. 2015), and GMAL2473 (*Rvi15* donor) were used as positive controls. DNA from 'Gala' served as negative control. Each sample was investigated in triplicate. Ratios were calculated from the Ct values following the assessment of target-specific efficiency using a dilution series (1:10) in triplicate, applying the formula for relative quantification (Pfaffl 2001).

### Insertion site determination and verification of the T-DNA-genome junctions

The insertion sites were determined by targeted locus amplification (TLA) technology. High-molecular-weight DNA of the cisgenic lines was extracted according to the protocol “Preparing Arabidopsis Genomic DNA for Size-Selected ~ 20 kb SMRTbell^™^ Libraries (PacBio SampleNet)”, pooled, and delivered to Cergentis (now Solvia NL, Utrecht, The Netherlands). TLA analysis was performed using primers specifically binding to portions of the T-DNA after excision (Suppl. Table S2). Sequences flanking the insertion sites were provided by Cergentis and mapped to the apple genome GDDH13 (Daccord et al. 2017) to identify the location of the insertion site in the genome. The genomic sequences flanking the insertion site were used to design specific primers (Suppl. Table S3), which were combined with primers on the T-DNA for PCR amplification of the T-DNA-genome junctions from DNA extracted from young apple leaves as described above. PCR was performed using the HotStarTaq PCR kit (Qiagen, Hilden, Germany) following the manufacturer’s protocol, in a 10 µl reaction with 2 µl DNA and an annealing temperature of 58 °C. PCR products were separated on a 1% agarose gel (80 V, 40 min) and visualized with ethidium bromide to confirm successful amplification. Amplicons were subsequently purified using the Multiscreen PCR 96 purification plate (Millipore, Burlington, USA), and Sanger sequenced (ABI 3130xl sequencer, Thermo Fisher Scientific Inc., Waltham, USA). Sequences were aligned and compared to the expected T-DNA structure and the genomic sequences at the insertion in the untransformed background ‘Gala Galaxy’.

### Fire blight inoculations

Three independent inoculation experiments were performed using 8–12 grafted plantlets of the cisgenic lines c47, c49, and c53, and ‘Gala Galaxy’ as susceptible control, grafted on M9 T337 rootstocks. Prior to inoculation, plantlets were grown for 4–6 weeks in the greenhouse until they reached the 6–8 leaf stages. Inoculations followed the protocol of Peil et al. (2007): the two youngest leaves of each plantlet were cut with scissors that had been dipped in a suspension of *E. amylovora* strain *EA222_JKI* at a concentration of approximately 2 billion bacteria per milliliter (OD_600nm_ = 1.03–1.05 in phosphate-buffered saline, PBS). The percentage of lesion length was recorded 21 days post-inoculation.

### Apple scab inoculations

Evaluation of apple scab resistance of the *Rvi15* cisgenic lines c603, c605, and c606, together with the resistant control GMAL2473 and the susceptible reference ‘Gala’, was performed on grafted greenhouse-grown plantlets following a protocol by Gianfranceschi et al. (1996). Prior to inoculation, plantlets were grown for 4–6 weeks until they reached the 6–8 leaf stage. Young leaves of at least two actively growing plants per genotype were sprayed with a suspension of *V. inaequalis* (concentration of approximately 3–5 × 10^5^ conidia/mL) and kept at 18 °C and 100% humidity in a tent for 48 h. Then, plants were removed from the inoculation tent to a greenhouse with ~ 70% relative humidity and evaluated 21 days after inoculation. The apple scab resistance tests were repeated four times, with a minimum of three shoots per genotype inoculated in each repetition.

## Results

### Apple scab-resistant cisgenic apple lines carrying *Rvi15*

**Fig. 1:**
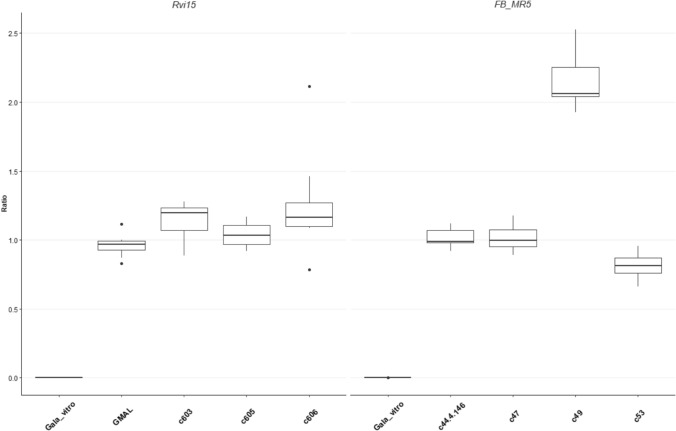
Estimation of the number of T-DNA insertion events in the new cisgenic apple lines. Boxplot representation of the qPCR-calculated copy-number ratios of *Rvi15* (left) and *FB_MR5* (right) in cisgenic lines, compared to genotypes carrying a single copy of the corresponding gene (GMAL2473 for *Rvi15* (Galli et al. 2010) and C44.4.146 for *FB_MR5* (Kost et al. 2015)). The untransformed cultivar ‘Gala’ (Gala_vitro) served as a negative control. Data were generated using three biological replicates, each tested in technical triplicates

In vitro cultures of the transgenic apple lines carrying *Rvi15* (t603, t604, t605, and t606) were provided from Schouten et al. (2014). None of the four transgenic lines was found to carry pMF1 backbone sequences adjacent to the T-DNA LB (data not shown). The cisgenic lines were regenerated from these transgenic lines via an induction step in which transgenic explants are cultured on a dexamethasone-amended medium that induces R recombinase activity (Vanblaere et al. 2011). The recombinase excises the cassette carrying the transgenes, as confirmed by PCR for the absence of the *NptII* gene (data not shown). Copy-number quantification via qPCR estimated a single integration of the *Rvi15* gene in lines c603, c604, c605, and c606 (Fig. 1).

**Fig. 2:**
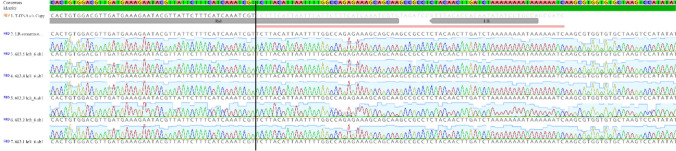
Alignments of sequences from the re-sequenced T-DNA-genomic junctions at the left border extremity of five cisgenic sublines (c603.1–5) derived from t603. The sublines were regenerated independently following excision of the transgenic cassette in the transgenic line t603. Chromatograms are shown with each sequenced sample as well as a consensus sequence (1^st^ element). The T-DNA-genome junction sequences were aligned to a sequence of the expected structure at the LB, with a red bar indicating T-DNA LB trimming and partial loss of the Rs recombination site. A vertical line indicates the junction between the remaining T-DNA extremity (left) and the adjacent genomic sequence (right)

The insertion sites were successfully identified in each line using TLA. TLA is a targeted sequencing approach that specifically sequences the cisgenic event and its flanking genomic DNA using primers designed on the cisgenic sequence and proximity ligation of DNA fragments near the cisgenic insertion event. Lines c603 and c604 share the same T-DNA insertion site on linkage group (LG) 16, indicating that the originating transgenic lines t603 and t604 were not independent events. Consequently, c604 was excluded from further analyses. Resequencing of the T-DNA genomic junctions at the LB extremity in five independently regenerated cisgenic sublines (c603.1–5) from t603 revealed identical sequences (Fig. 2), confirming the reproducibility of recombinase-based cassette excision.

In lines c605 and c606, the T-DNA was inserted on LG 5 and LG 14, respectively (Table 1). For line c606, no sequences adjacent to the T-DNA RB could be retrieved. Amplification and sequencing of the T-DNA-genomic junctions of c603 and c605 revealed near-complete trimming of the RB, leaving up to 73 bp of non-endogenous sequence between the RB and *Rvi15* 5’-UTR. Similarly, trimming of 34–60 bp was observed at the opposite T-DNA extremities in c603, c605, and c606, leaving up to 92 bp of non-endogenous sequence, mainly consisting of the 81 bp recombinase recognition sequence Rs. T-DNA integration caused deletions of 40 bp and 285 bp of genomic sequences in lines c603 and c605, respectively.

**Table 1:** Overview of the T-DNA integration characteristics in the cisgenic lines generated in this work

Cisgenic line	Resistance gene	Chromosome (GDDH13, Daccord et al. 2017))	Genome position adjacent to the RB	Genome deletion or insertion	Genome position adjacent to the LB	Trimming at the RB extremity	Vector-based sequences left at the RB extremity	Trimming at the LB extremity	Rs -trimming	Vector-based sequences left at the LB (Rs) extremity	Vector-based sequences left total (sum of RB and LB)
c603	*Rvi15*	16	12,990,850	−33 bp	12,990,883	26 bp	72 bp	71 bp	33 bp	63 bp	135 bp
c605		5	45,112,657	−287 bp	45,112,370 (3’-UTR of MD05G1324300)	25 bp	73 bp	34 bp	4 bp	89 bp	162 bp
c606		14	NA	NA	21,235,991	NA	NA	42 bp	5 bp	76 bp	NA
c47	*FB_MR5*	11	15,386,015	−64 bp	15,385,952	22 bp	38 bp	36 bp	6 bp	133 bp	171 bp
c49 (LB chr08, RB chr09)		8 + 9	16,358,582	+ 8 bp (chr8, RB)	33,131,773	22 bp	38 bp	6 bp	0 bp	163 bp	201 bp
c53		NA	n.i	n.i	n.i	NA	NA	NA	NA	NA	NA

The table includes the location of the insertion site according to the GDDH13 apple reference genome assembly, genomic deletions/insertions at the right border (RB) and left border (LB), and residual vector-derived sequences of each line

**Fig. 3:**
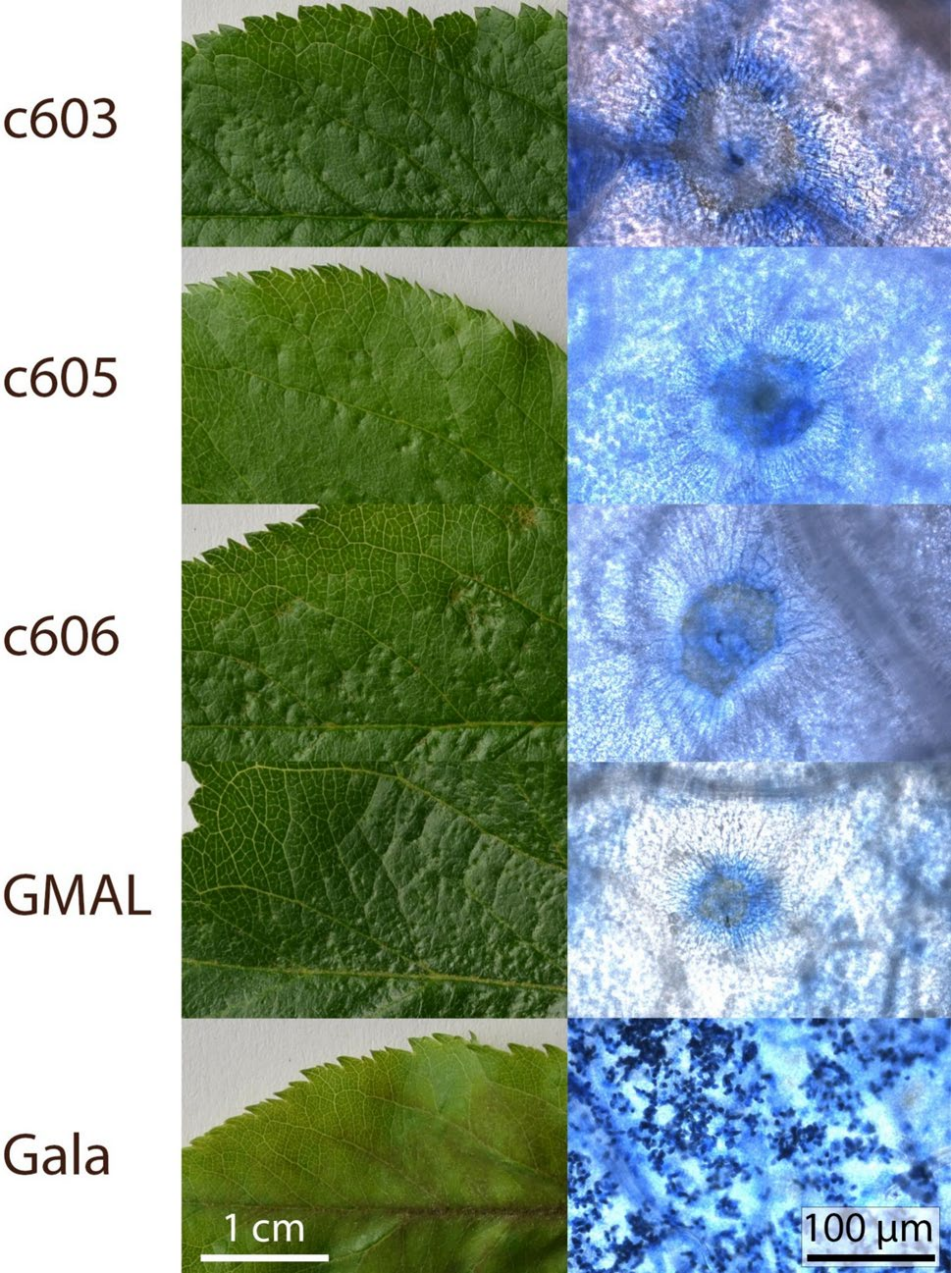
Macroscopic and microscopic responses of cisgenic apple lines and controls to *Venturia inaequalis* infection. Representative macroscopic (left) and microscopic (right) images of apple leaf responses to *V. inaequalis* inoculation in cisgenic lines carrying *Rvi15* (c603, c605, and c606), the resistance donor GMAL2473 (GMAL), and the susceptible control ‘Gala’ 21 days post-inoculation (dpi). Scale bars are directly indicated in the image. Hypersensitive reactions (pinpoint pits) were observed in all *Rvi15* lines and the donor, while only the susceptible control ‘Gala’ displayed sporulation. Leaves were stained for microscopy imaging according to Galli et al. (2010). Representative pictures were selected from four experimental repetitions with at least two leaves per genotype per inoculation experiment

In all inoculation experiments with *V. inaequalis* in the greenhouse, the *Rvi15* cisgenic lines, as well as the resistance donor GMAL2473, reacted with a hypersensitive defense response (pinpoint pits), the typical defense reaction of *Rvi15* genotypes, while the untransformed genotype ‘Gala Galaxy’ showed sporulation (Fig. 3). No sporulation and pinpoint pits were observed on all the three cisgenic lines c603, c605, and c606, confirming the apple scab resistance conferred by *Rvi15*.

### Fire blight-resistant cisgenic apple lines carrying *FB_MR5*

Three independent *Agrobacterium*-mediated transformations were performed using the pMF1::*FB_MR5* vector using 614 ‘Gala Galaxy’ leaf explants. A total of 32 transgenic lines were regenerated, of which 7 showed backbone integration as indicated by positive PCR amplification (data not shown). Three transgenic lines (t47, t49, and t53) were selected for excision of the transgenic cassette, resulting in cisgenic lines c47, c49, and c53. qPCR targeting *FB_MR5* indicated a single insertion in the lines c47 and c53, while the cisgenic line c49 carried two insertions (Fig. 1).

TLA confirmed the number of insertions and identified the insertion site of *FB_MR5* on chromosome 11 for c47 and on chromosomes 8 and 9 for c49. No insertion site could be retrieved for c53. As in *Rvi15* lines, T-DNA extremities trimming was observed. Non-endogenous sequences of 38 bp at the RB and up to 163 bp at the LB were detected (Table 1, Suppl. Fig. S2).

**Fig. 4:**
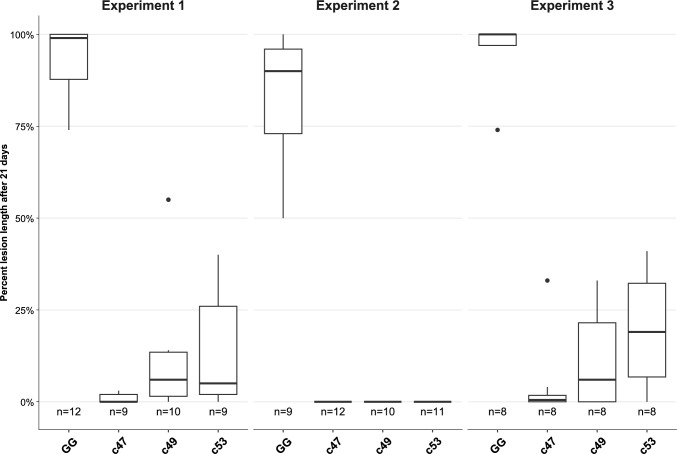
Boxplot representation of the percent lesion length 21 days after inoculation with *E. amylovora*. Cisgenic lines c47, c49, and c53 carrying *FB_MR5* are compared to the control ‘Gala Galaxy’ (GG). *n:* number of inoculated shoots

In three independent shoot inoculation experiments with *E. amylovora*, all cisgenic lines c47, c49, and c53 showed significantly reduced symptom severity, measured as percentage lesion length three weeks after inoculation, compared to the highly susceptible wild-type ‘Gala Galaxy’ (Fig. 4).

## Discussion

This study demonstrates the successful application of the cisgenic approach to improve the resistance in the commercial apple cultivar ‘Gala’ against two major diseases in apple orchards, apple scab and fire blight. The disease resistance levels attained were high and comparable to those reported in earlier studies investigating the same resistance genes *FB_MR5* and *Rvi15* (Kost et al. 2015; Schouten et al. 2014). Through *Agrobacterium*-mediated transformation, T-DNA carrying either *Rvi15* or *FB_MR5* was inserted predominantly as single copies in the majority of cisgenic lines. TLA successfully identified insertion sites in two of three cisgenic lines per resistance gene, confirming random T-DNA integration on different chromosomes, and consistent with qPCR-based copy-number estimates. Sequences on both ends of the T-DNA were trimmed during the insertion process, as previously reported by others (Vanblaere et al. 2014, Kost et al. 2015, Dalla Costa 2020). Recombination-driven excision of the transgenic cassette in line t603 was successful, although not only the LB sequence but also approximately one-third of the restriction recombination site was removed. Resequencing of five cisgenic sublines obtained from five independent recombination events from t603 confirmed that recombination worked consistently with no sequence variation among the sublines. The length of vector-derived sequences remaining in the final cisgenic products using the pMF1 vector ranged between 38 and 73 bp at the RB and from 63 to 163 bp on the LB. These numbers are in line with those reported previously for pMF1 (Vanblaere et al. 2014) and are lower than the vector-based sequences observed using the p9-Dao-FLPi vector. For example, in line C44.4.146, 94 bp and 359 bp of vector-derived sequences were retained at the RB and LB, respectively (Kost et al. 2015). As long as the development of cisgenic lines depends on a recombinase requiring recognition sequences, at least one copy of the recombination site (34 bp for FRT, 81 bp for Rs) will remain in the final product. Dalla Costa et al. (2020) proposed using an inducible CRISPR/Cas system to avoid recombinase recognition sequences. However, the authors reported that even this strategy would leave a minimum of 37 bp of non-endogenous sequences in the final product (Dalla Costa et al. 2020).

Non-endogenous sequences, whether vector-derived or transgenic, may remain in the final product and raise a regulatory concern. If regulators establish a threshold for such sequences based on their length, cisgenic plants could be categorized alongside conventional GM plants or exempted from certain parts of the authorization assessment process. Another potential criterion for exemption could be the precise targeted insertion of the cisgene into a specific genomic locus. However, achieving cisgenic plants free of non-endogenous sequences and ensuring targeted insertion often present a conflict, as non-endogenous sequences, as Lox66 or FRT1 recombination sites, are necessary to facilitate cisgene insertion by e.g., Primeroot editing technology (Sun et al. 2024) and are left in the final product.

Our previous research showed that the tissue culture process itself can induce unintended phenotypic deviations. Specifically, the wild-type genotype ‘Gala Galaxy’ used to generate cisgenic line C44.4.146 (and the *FB_MR5* lines described here) had altered fruit coloration with reduced red overcolor (Schlathölter et al. 2023). This highlights the importance of monitoring somaclonal variation, especially in cultivars whose desirable traits may rely on mutations in specific meristematic layers (Foster & Aranzana 2018). It is important to assess if such phenotypic deviations also occur in different in vitro lines of the same cultivar.

To improve the durability of the disease resistance achieved by cisgenesis, and similarly to the approaches used in conventional breeding, several resistance genes targeting the same disease should be deployed in pyramids within the same genotype (Kellerhals et al. 2009). *FB_MR5*, *Rvi15*, *Rvi12*, and *Rvi6* have limited durability, as shown by the emergence of virulent pathogen strains that overcome these resistances (Emeriewen et al. 2018; Parisi et al. 1993; Parravicini et al. 2011; Patocchi et al. 2020; Vogt et al. 2013). Additional apple scab resistance genes should be identified in the near future, as their cloning is ongoing (Nadeesha Lewke et al. 2019). For fire blight, *FB_MR5* remains the only cloned gene to date, but the cloning of *FB_E* from ‘Evereste’, and *FB_Fu* from *Malus fusca* was initiated (Emeriewen et al. 2018, 2021; Parravicini et al. 2011). In conclusion, combining strategies to ensure resistance durability through gene pyramiding, minimizing non-endogenous sequences, and ensuring the phenotypic stability of the in vitro plant material will allow the development of cisgenic lines with high fruit quality and durable disease resistance, contributing to the reduction of plant protection product use in apple production and avoiding yield losses.

## Supplementary Information

Below is the link to the electronic supplementary material.

Supplementary file1 (DOCX 3208 KB)

## Data Availability

All data generated or analyzed during this study are included in this published article and its supplementary information files.
