# Supplementary material for: Generation of new cisgenic apple lines resistant to either apple scab or fire blight
Source: Planta. 2026 Jun 24;264(2):28. doi: 10.1007/s00425-026-05051-6 (PMC13294234; doi:10.1007/s00425-026-05051-6)
Supplement: Supplementary file 1 — Supplementary file1 (DOCX 3208 KB) [file 425_2026_5051_MOESM1_ESM.docx]

Supplementary information

**
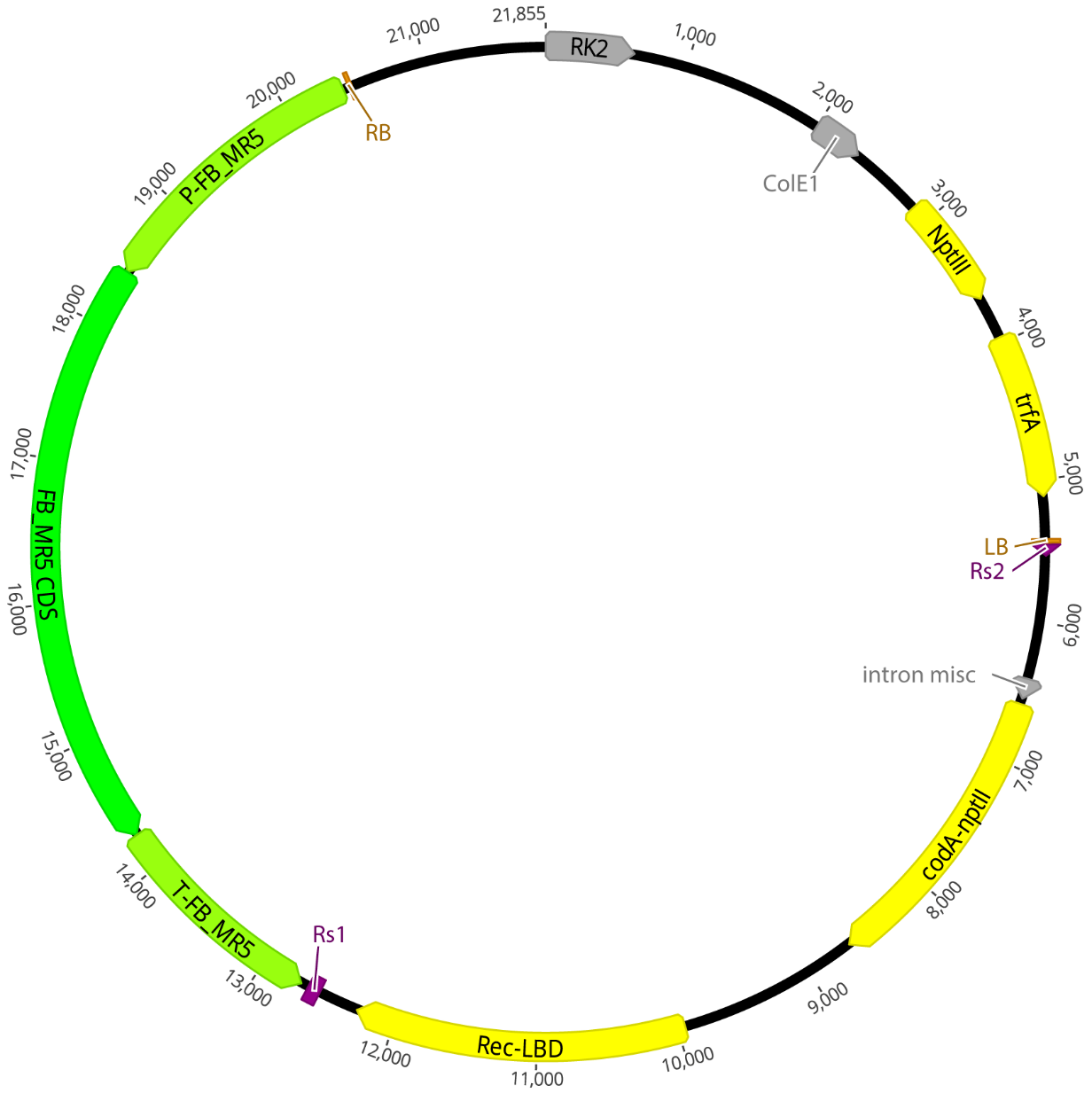
**

**Suppl. Fig. S1** Map of the pMF1::*FBMR5* vector used for the generation of fire blight resistant cisgenic apple lines. The fire blight resistance gene *FB_MR5* with native coding elements (P Promoter, T Terminator CDS coding sequence) is colored green. The segment between the left (LB T-DNA repeat) and the right border (RB T-DNA repeat) is transferred into the plant cell, and the segment containing *codA-nptII* and *Rec-LBD* between the recombination sites (Rs1 and Rs2) is then removed on recombinase-mediated excision. Marker gene *codA-nptII*, hybrid gene for positive (*NptII*) and negative (*codA*) selection. *Rec-LBD*, translational fusion of recombinase *R-LBD*; RK2 and ColE1, origins of replication. *trfA*; replication gene; *nptIII*, kanamycin resistance gene


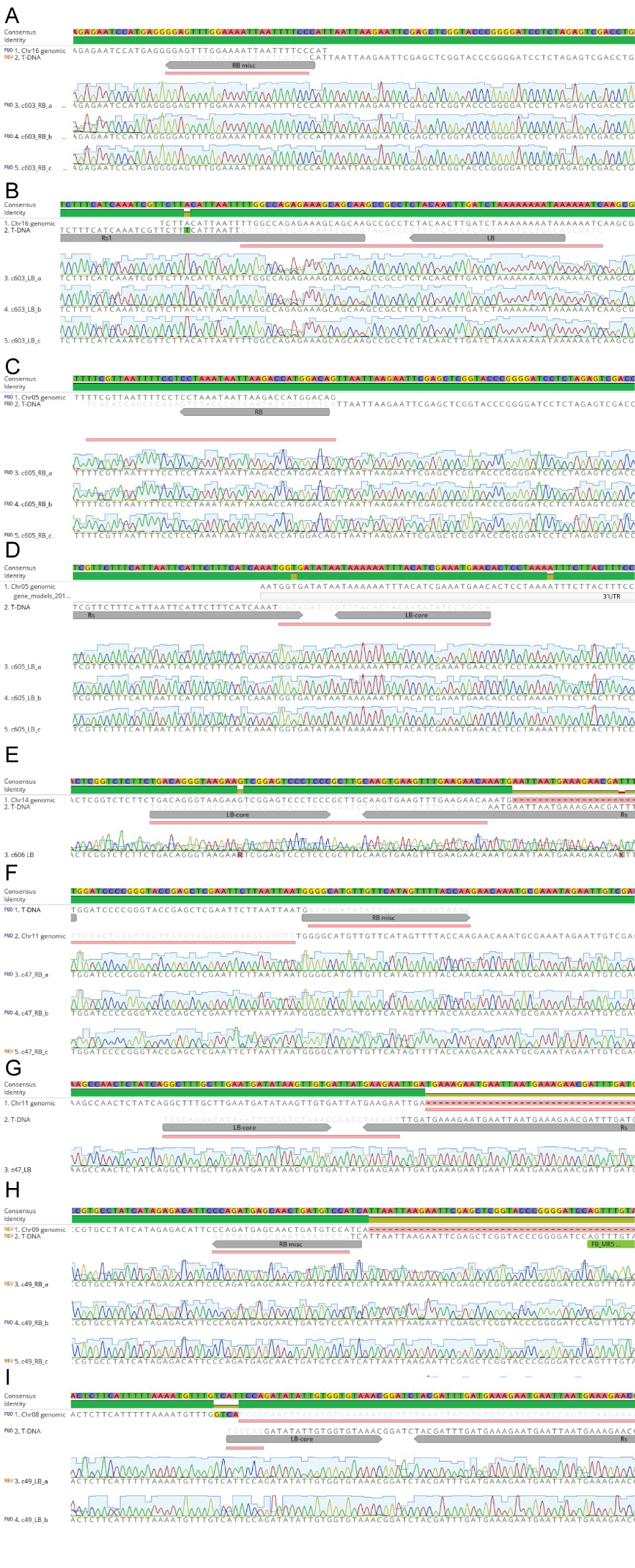


**Suppl. Fig. S2** Sequence alignments of the T-DNA integration junctions in the investigated cisgenic lines. Sequence alignments of chromatograms from the re-sequenced T-DNA-genomic junction sequences of the PCR products from investigated lines were aligned to a concatenated reference sequence composed of the T-DNA structure of the corresponding transformation vector and the genomic region at the insertion site (2^nd^ element in each picture). A red bar under the sequence highlights either trimmed regions at the T-DNA borders or segments of the genomic sequence that were omitted to improve alignment clarity. (A) RB of c603, (B) LB of c603, (C) RB of c605, (D) LB of c605, (E) LB of c606, (F) RB of c47, (G) LB of c47, (H) RB of c49, and (I) LB of c49.

**
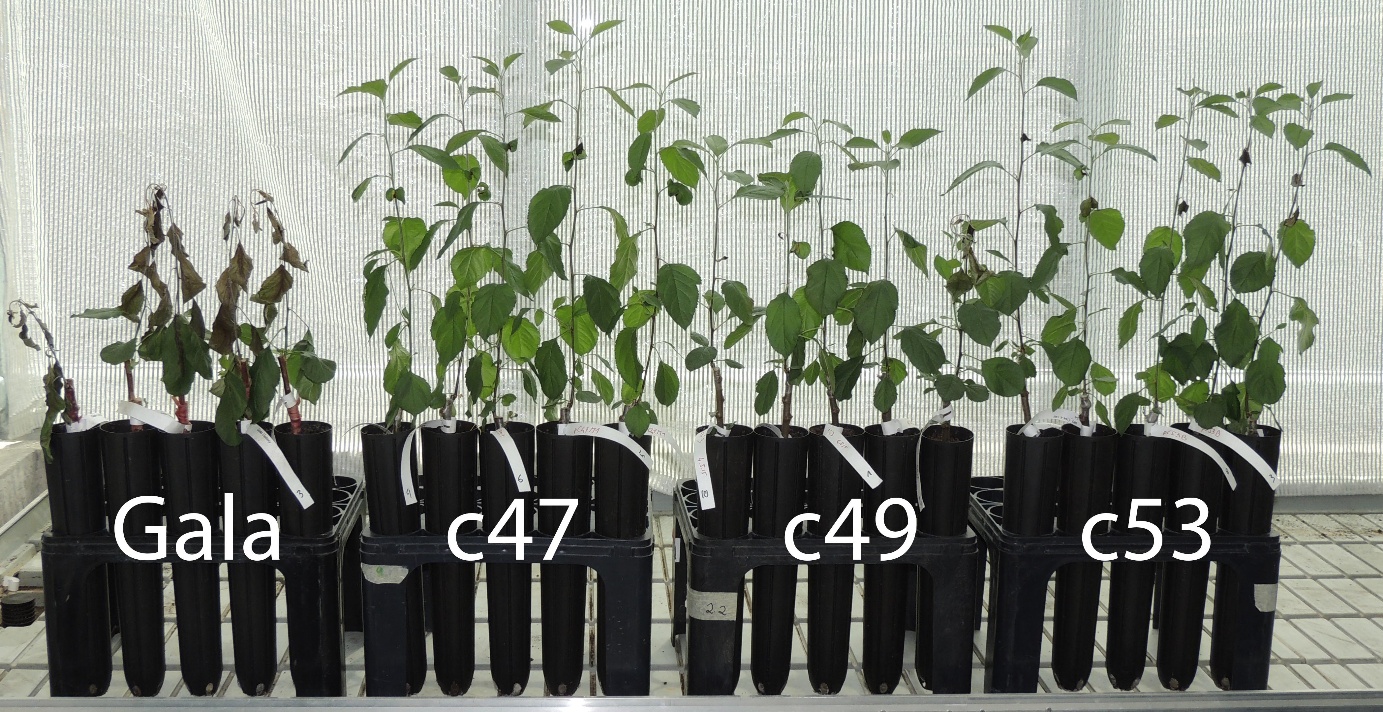
**

**Suppl. Fig. S3** Representative photograph showing five representative plants per genotype, including Gala Galaxy and cisgenic lines c47, c49, and c53 carrying the *FB_MR5* fire blight resistance gene, 21 days after scissor-mediated inoculation with *Erwinia amylovora strain EA222_JKI*. For size reference in the image, the plant tubes are 35.5 cm tall.

**Suppl. Table S1** *Rvi15* TaqMan assay primers and probe

| **Name** | **5’-3’ sequence** | **Final conc.** | **Modifications** |
| --- | --- | --- | --- |
| Rvi15q1 forward | ATCTCGGACAGATTAATACAG | 900 nM |  |
| Rvi15q1 reverse | TGAAAACCCTTGATCTCAGCTGG | 900 nM |  |
| Rvi15 TaqMan Probe | YY-TTT57C7AGT5CC7A7T5T-BHQ1 | 250 nM | 5 = pdC  7 = 2AdA  YY = Yakima Yellow  BHQ1 = Black Hole Quencher 1 |

**Suppl. Table S2** Primer sequences designed for TLA according to the corresponding disease resistance gene present on the pMF1 vector

| **Name** | **5’-3’ sequence** | **Used for TLA of the cisgenic lines carrying following resistance gene** |
| --- | --- | --- |
| RV_7735 | AACGATTGATTACACGGTCT | *FB_MR5* |
| TG:106 | CAGTGATAGGATCTGGCG | *FB_MR5* |
| FW_106/TG:106 | CAGTGATAGGATCTGGCG | *Rvi15/FB-MR5* |
| RV_115 | CTTATTCGCGCGCCAGATC | *Rvi15* |
| TG:10,451/RV_10432 | GATCCTCTAGAGTCGACCTG | *Rvi15* |
| RV_82 | GGACGTTGATGAAAGAATACGTT | *FB_MR5* |
| FW_7889/TG:10,457/TG:7,889 | GTACCGAGCTCGAATTCTTA | *Rvi15/FB-MR5* |
| FW_10457 | GTACCGAGCTCGAATTCTTA | *Rvi15* |
| FW_7848 | TCATCGAATTAGTCTGTGTAGT | *FB_MR5* |
| TG:47 | TCTTTCATCAAATCGTAGATCC | *FB_MR5* |
| TG:47 | TCTTTCATCAAATCGTAGATCC | *Rvi15* |
| TG:7,748 | TGATTACACGGTCTTAAAGGT | *FB_MR5* |

**Suppl. Table S3** Primer sequences designed on T-DNA-genomic junctions

| **Name** | **5’-3’ sequence** | **LB/RB** | **Line** | **T-DNA/**  **genomic** |
| --- | --- | --- | --- | --- |
| Vr2-cDNA_10144 | TCTACTAGTGATGCTGCAGGT | LB | c603/4 | T-DNA |
| chr16_10630 | TGGCAACCAGAATTGACTCACT | LB | c603/4 | genomic |
| chr16_82_F | TCGGCTCAATTAGGTGTAATT | RB | c603/4 | genomic |
| Vr2-cDNA_525 | TGTTGTCCACATGCACTTGAG | RB | c603/4 | T-DNA |
| chr5_GDDH_45113259R | TGCCCTCTTGTTTGTGTGGT | RB | c605 | genomic |
| Vr2_RB-510F | GGTTGGTGGAGAGCAGTTCA | RB | c605 | T-DNA |
| Vr2_LB-581R | TCAATGAACTCGTTTGCGCA | LB | c605 | T-DNA |
| chr5_GDDH_45112167F | TTTCATTTGAATTCATTTTAACCCCC | LB | c605 | genomic |
| Vr2_LB-353R | TCTACTAGTGATGCTGCAGGT | LB | c606 | T-DNA |
| chr14_21235558F | GTTGGATGAGCGGATGCAGA | LB | c606 | genomic |
| chr11_17013767_F | TGCTAAAGGGCAAGTGCGA | LB | c47 | genomic |
| C44.4.146_reseq915_R | ACGGTGAAAGGTGTGGACTT | LB | c47/c49 | T-DNA |
| C44.4.146_reseq8,203_F | GACCGTGTAATCAATCGTTTGCA | RB | c47/c49 | T-DNA |
| chr11_17014834_R | GCAAACCCAAGTGCACTGAG | RB | c47 | T-DNA |
| chr8_33131278F | TGACGTGGTCAAATTGGCTT | LB chr8 | c49 | genomic |
| chr9_16359091R | CGAGAAGTGTGAGAAGGCGT | RB chr9 | c49 | genomic |
